# In Situ Characterization of Damage Development in Cottonid Due to Quasi-Static Tensile Loading

**DOI:** 10.3390/ma13092180

**Published:** 2020-05-09

**Authors:** Ronja Scholz, Alexander Delp, Frank Walther

**Affiliations:** Department of Materials Test Engineering (WPT), TU Dortmund University, Baroper Str. 303, D-44227 Dortmund, Germany; alexander.delp@tu-dortmund.de (A.D.); frank.walther@tu-dortmund.de (F.W.)

**Keywords:** Cottonid, cellulose, in situ testing, scanning electron microscope, microfocus computer tomograph, quasi-static loading, microstructure, damage development, damage mechanisms, crack initiation

## Abstract

Cottonid is a layered material based 100% on cellulose that holds excellent material properties by being completely sustainable. The finite nature of petroleum-based resources nowadays makes these properties significant for technical applications again. To understand how Cottonid reacts to application-oriented mechanical loads and how it fails, development of microstructural damage on the surface and in the volume of Cottonid was studied using innovative in situ testing techniques for the first time. Quasi-static tensile tests were comparatively performed in a scanning electron microscope as well as a microfocus computer tomograph, and the development of defects present in the initial condition of the material was investigated. In the elastic region, no visible damage initiation on the surface and a decrease of overall void volume within the gauge length could be detected. When reaching the yield strength, crack initiation on the surface starts at critical areas, like pores and microcracks, which propagation and assembly could be visualized via scanning electron micrographs. In the plastic region, an increase in void volume could be shown in the gauge length until final failure of the specimen. Innovative material testing techniques presented in this study support lifetime estimation in technical applications and understanding of process–structure–property relations. Particularly, characterization of microstructural damage development due to a mechanical load, which leads to final failure of the specimen, is essential to be able to create material models for lifetime prediction in respect to variable manufacturing or application parameters.

## 1. Introduction

Cottonid is a cellulose-based polymer, which was developed in 1844 by J. Mercer and patented by T. Taylor in 1859. The material is manufactured by parchmentizing unsized filter paper layers using a zinc chloride (ZnCl_2_) or sulphuric acid (H_2_SO_4_) solution. The source of cellulose derivation for the paper making process could be cotton linters—also from textile waste—or wood pulp. In a tempered catalyst bath, new intra- and intermolecular hydrogen bonds are formed by etching the surfaces of the cellulose fibrils contained in the paper. The thickness of the resulting plate material is defined by the amount of paper layers fed into the process [1,2]. In the beginning of the 20th century, Cottonid was replaced by synthetic plastics in most technical applications and, despite the long awareness of the material, research activities have remained static over a long time. Therefore, the material can mostly be found in niche applications nowadays [3,4]. Today, with regard to sustainability and eco-friendliness, Cottonid is a resource-efficient alternative to conventional construction materials again, but the known material parameters were obtained following outdated standards, and Cottonid‘s damage development and failure mechanisms stayed unexploited since innovative testing equipment and strategies are needed for their detection.

First studies [5,6] addressed the quantitative mechanical properties of Cottonid. The material shows a direction-dependent deformation behavior due to a preferred orientation of the cellulose fibrils in manufacturing direction of the raw paper, i.e., highest mechanical strength (ultimate tensile strength, UTS) can be obtained in manufacturing direction and is comparable to common technical plastics, like polyamide (PA) or polyvinyl chloride (PVC), and wood-based materials [7]. Depending on the material thickness t_mat_ (amount of paper layers), Cottonid shows a more or less pronounced and directed swelling and shrinking behavior in reaction to varying relative humidity, similar to wood. These properties were investigated with respect to the chosen manufacturing parameters, with the aim to use Cottonid as a functional material in terms of climate-adaptive architectural applications [8,9,10].

The instrumentation of mechanical test setups with physical sensors for characterization of deformation and damage initiation and evolution during a mechanical loading is the state of the art for various materials [11,12,13]. Monitoring of material reactions, like deformation [14], change in temperature [15] or electrical resistance [16], and acoustic emissions [17,18], allows an assessment of the structural integrity and identification of occurring damage mechanisms of the tested specimen before final failure [19]. By combining mechanical testing with analytical techniques, like scanning electron microscopy (SEM) or microfocus computer tomography (µCT), an optical characterization of microstructural changes during loading can be realized and correlated with the macroscopic deformation behavior [20]. To evaluate load-induced damage in relation to the initial condition of the specimen, conventional SEM or µCT is not efficient, since when intermitting a macroscopic mechanical test to scan the specimen, occurred damage mechanisms might not be visible anymore in the unloaded structure [21]. In situ SEM [22,23] and µCT [24,25,26] investigations, on the other hand, enable the analysis of the microstructure in a loaded state, which leads to a basic understanding of effective damage mechanisms leading to final failure of the specimen [27]. The efficiency of high-resolution computed tomography techniques for visualizing the microstructure of low density polymeric materials, like Cottonid, has been shown in various studies on the example of fiber-reinforced structures [28,29] or wood tissue [30,31,32]. Furthermore, for interpretation of monitored microstructural changes due to mechanical loading, theories regarding the damage propagation in laminated [33,34], fiber-reinforced [35] and semi-crystalline [36] composite materials were used for orientation in this work.

After studies on the macroscopic deformation behavior of Cottonid in response to mechanical or hygroscopic loading, with this work, a first approach is made to investigate microstructural damage development and mechanisms of Cottonid exemplary for quasi-static tensile loading. To assess the microstructural changes during loading, qualitative and advanced optical surface and volume analyses via in situ SEM and µCT techniques were performed.

## 2. Materials and Methods

### 2.1. Sample Preparation

**Figure 1:**
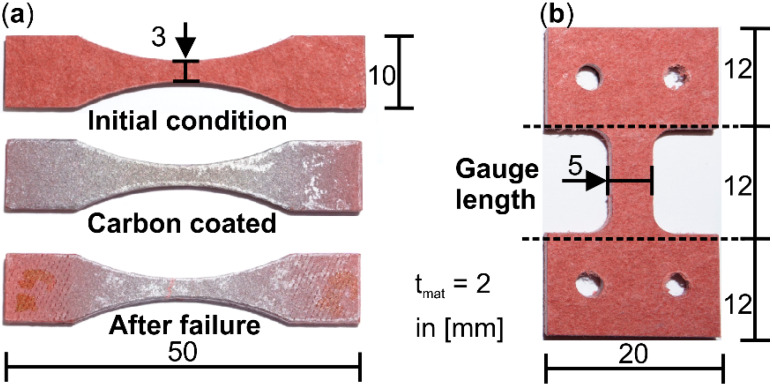
Specimen geometry for in situ tensile tests in (**a**) scanning electron microscope (SEM); and (**b**) microfocus computer tomograph (µCT).

The specimens for the following investigations were milled out of a plate of industrial Cottonid material (Ernst Krüger GmbH & Co. KG, Geldern, Germany) with a thickness of t_mat_ = 2 mm and were trimmed and cleaned afterward using compressed air. For in situ investigations in a SEM, specimens were additionally coated with carbon under high vacuum conditions (208 Carbon, Cressington). Geometries were chosen in respect to the assembly dimensions of the in situ testing machines (Figure 1). For conditioning, specimens were stored under laboratory conditions (temperature T = 23 ± 2 °C, relative humidity φ = 35 ± 5%) for a time t > 48 h before testing.

### 2.2. Test Setup for Microstructural In Situ Investigations

**Figure 2:**
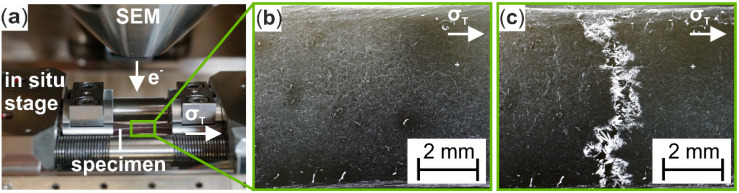
In situ tensile test in SEM: (**a**) integrated micro-tension module (Kammrath & Weiss GmbH, Dortmund, Germany) with mounted Cottonid specimen; SEM micrographs of gauge length’s surface (**b**) in initial condition; and (**c**) after failure.

For microstructural investigations on the surface of Cottonid specimens (Figure 1a), a field emission scanning electron microscope (FE-SEM, MIRA 3, Tescan GmbH, Dortmund, Germany) was used. Beam voltage was set at UB = 10 kV, while magnification M settled between M = 50 and 5 × 10^3^. Tensile loading during surface observation with SEM was applied with a testing speed of v = 0.12 mm/min using a micro-tensile testing module (F_max_ = 5 kN, Kammrath & Weiss, Dortmund, Germany). Figure 2a shows the test chamber of the SEM with the integrated in situ module and the mounted Cottonid specimen. Furthermore, exemplary SEM micrographs of the specimen’s surface in different deformation stages (Figure 2b,c) are given to illustrate the in situ testing technique for characterization of damage development.

**Figure 3:**
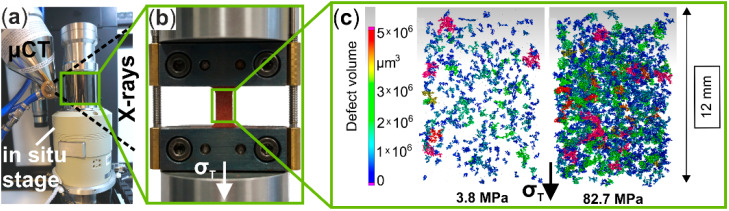
In situ tensile test in µCT: (**a**) integrated micro-tension/compression module (CT5000 GCT-RT, Deben Dortmund, Suffolk, UK); (**b**) the mounted µCT specimen; and (**c**) void volume in the specimen’s gauge length at two exemplary tensile stresses.

Microstructural investigations in the volume of the specimen’s gauge length (Figure 1b) were performed in a microfocus computer tomograph (µCT, X TH 160, Nikon Metrology GmbH, Alzenau, Germany) with maximum beam energy of U_max_ = 160 kV and maximum power of P_max_ = 60 W. Analogous to SEM investigations, a micro-tensile/compression testing module (F_max_ = 5 kN, 5000CTGCT-RT, Deben UK Ltd., Suffolk, UK) was used for in situ tensile tests during a µCT scan. Figure 3a shows the test chamber of the µCT with the integrated in situ module and Figure 3b shows the mounted Cottonid specimen. Furthermore, exemplary void volumes in the specimen’s gauge length in different deformation stages are shown (Figure 3c). Scanning parameters for the µCT scans were U_scan_ = 125 kV and P_scan_ = 6.4 W resulting in an effective pixel size of 7 µm. Testing speed was v = 0.5 mm/min. During one scan, 1583 projections were captured, each with an exposure time of t_ex_ = 250 ms. The obtained data were reconstructed and post-processed with industrial CT software (VG studio max V.2.2, Volume Graphics GmbH, Heidelberg, Germany) via threshold defect analysis.

For characterization of damage development in Cottonid due to quasi-static tensile loading, a comparative evaluation of the microstructure on the surface and in the volume at different load steps was performed in comparison to the initial condition of the specimens. The obtained SEM micrographs were therefore analyzed for characteristic defects, like pores or microcracks, which could be monitored during the in situ test. On the reconstructed 3D volumes from µCT scans, threshold defect analyses for comparison of void volumes in the specimens‘ gauge lengths were applied.

### 2.3. Test Strategy for Characterization of Damage Development on the Surface and in the Volume

**Figure 4:**
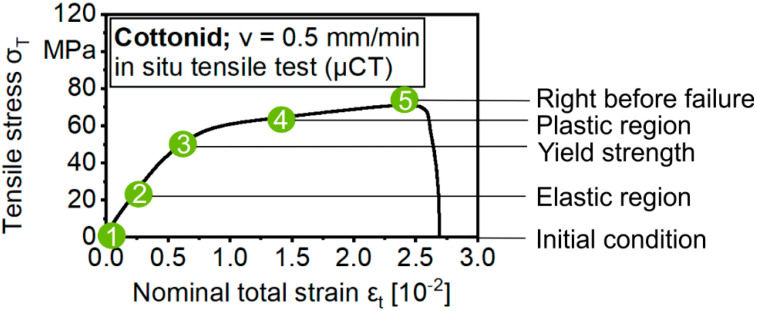
Determination of significant regions in the stress–strain behavior of Cottonid to visualize damage development on the surface and in the volume.

Characteristic load steps, where SEM micrographs and 3D volumes should be taken, were determined on the basis of the stress(σ)–strain(ε) behavior of Cottonid when applying quasi-static tensile loading under laboratory conditions (Figure 4). Significant microstructural changes due to mechanical loading in comparison to the initial condition (1) of the specimen were expected in the elastic region (2), at the yield strength (3), in the plastic region (4) and finally at the point right before failure (5).

For SEM investigations, a testing speed of v = 0.12 mm/min was chosen to minimize relaxation effects during the hold times in in situ tensile testing because of the visco-elastic deformation behavior of the polymeric material Cottonid. After adjusting the SEM parameters and clarifying the region of interest, three tests were conducted in sum by simultaneously taking SEM micrographs in the predefined regions of the σ–ε curve (Figure 4).

**Figure 5:**
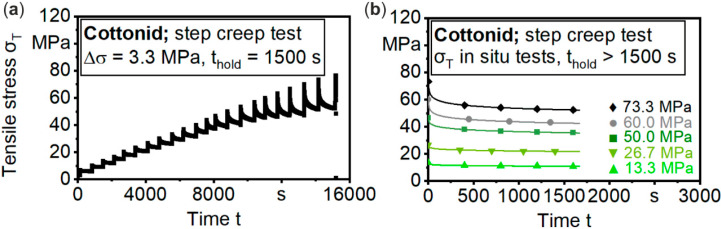
Assessment of relaxation behavior (**a**) stepped creep test; and (**b**) single creep tests for different tensile loads with functional fit.

To minimize relaxation effects, before starting a µCT scan, a lead time had to be determined to avoid image artifacts in the projections while keeping up a constant nominal strain during a total scanning time of t_scan_ > 90 min. To assess the relaxation behavior of Cottonid at different tensile loads, step creep tests with load steps of Δσ = 3.33 MPa and a hold time at each step of t_hold_ = 15 min (Figure 5a), as well as step creep tests at discrete tensile loads on the basis of the predefined characteristic regions of the σ–ε curve (σ_1_ = 13.33 MPa; σ_2_ = 26.66 MPa; σ_3_ = 50 MPa; σ_4_ = 60 MPa; σ_5_ = 73.3 MPa) (Figure 5b), also with a hold time of t_hold_ = 15 min each, have been performed.

By fitting the obtained curves during t_hold_ with a power function (R^2^ ≈ 0.99), an empiric two-stage rule for tensile stresses σ_T_ < 30 MPa and σ_T_ ≥ 30 MPa could be developed with Equations (1) and (2):

σ_T_ = σ_s_·t^−0.032^ for 0 MPa < σ_s_ < 30 MPa (1)

σ_T_ = σ_s_·t^−0.048^ for σ_s_ ≥ 30 MPa (2)

where t (s): time, σ_T_ (MPa): tensile stress, and σ_s_ (MPa): targeted tensile stress in in situ test.

Taking into account predefined test criteria, like a maximal relaxation of 0.4 MPa/min (equivalent to 0.1 N/s with chosen geometry) and 3.33 MPa/scan with a total duration of t_scan_ > 90 min, respectively, a lead time before starting a µCT scan was calculated for each load step using Equations (1) and (2) (Table 1). Three specimens were tested for each step creep test variation. In the main investigations, three specimens were tested for each predefined load step (Figure 4).

## 3. Results

### 3.1. In Situ Quasi-Static Tensile Tests in SEM

**Figure 6:**
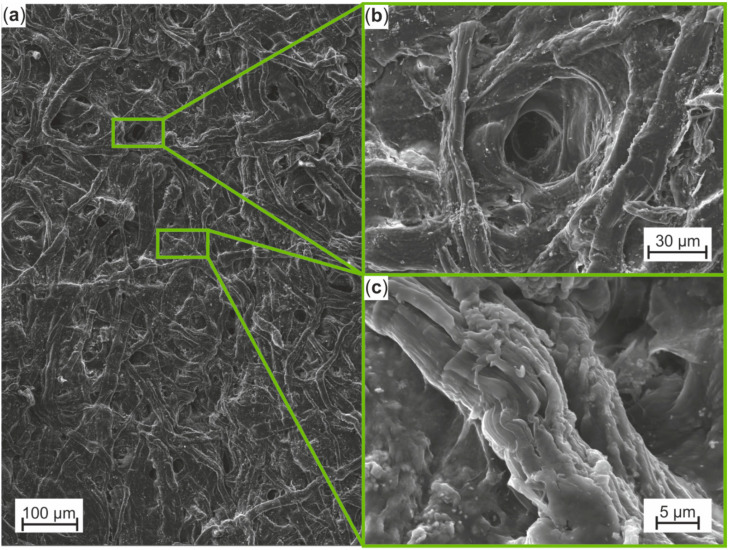
SEM micrographs of Cottonid’s surface for identification of characteristic surface defects in initial condition: (**a**) field of view in in situ test; (**b**) pore; and (**c**) single fiber.

Figure 6a visualizes the surface of the gauge length of the SEM in situ specimen in initial condition. It consists of bound and partly bound cellulose fibers, pores and amorphous areas, which could be identified as significant microstructural characteristics to visualize damage development due to quasi-static tensile loading. The close ups of a pore and a single fiber (Figure 6b,c), showing its waviness, reveal the imperfection of the surface of Cottonid, even in initial condition.

**Figure 7:**
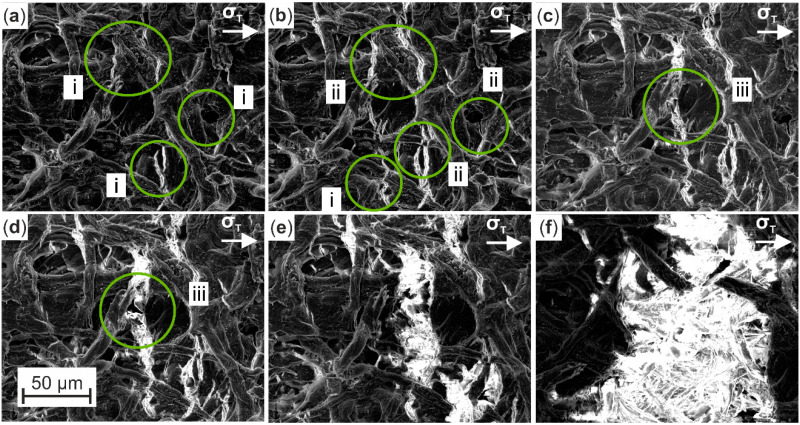
Exemplary in situ SEM monitoring of development of surface defects (i: crack initiation, ii: crack propagation, iii: crack assembly) due to increasing tensile loading of the specimen until final failure: (**a**) crack initiation (i) at critical areas; (**b**) crack propagation (ii) throughout the whole structure; (**c**,**d**) crack assembly (iii); (**e**) complete loss of structural integrity; (**f**) final failure.

An exemplary monitoring of predefined microstructural surface characteristics is presented in Figure 7. By observing the micrographs at increasing tensile loads, occurring damage mechanisms, like crack initiation and propagation, were distinguished and marked, so that their development and proportion to final failure of the specimen could be characterized throughout the in situ test. The identified imperfection of Cottonid’s surface leads to crack initiation (Figure 7a, i) at critical areas, like pores or already existing microcracks. Cracks propagate (Figure 7b, ii) with increasing tensile stress σ_T_ throughout the whole structure by cutting cellulose fibers and assembling (Figure 7c,d, iii), which leads to a complete loss of structural integrity (Figure 7e) and final failure (Figure 7f) of the specimen.

**Figure 8:**
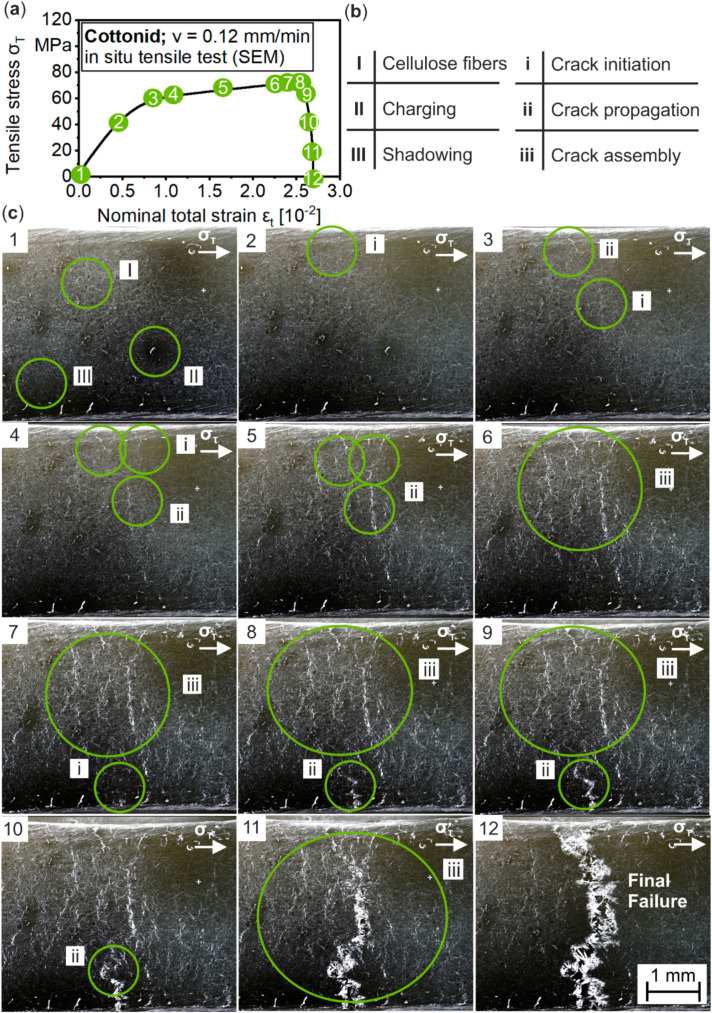
In situ tensile test in SEM: (**a**) significant points in stress–strain curve for obtaining SEM micrographs; (**b**) labelling of characteristic surface areas and occurring damage mechanisms; and (**c**) visualization of damage development on the surface of Cottonid due to quasi-static tensile loading.

Following this procedure and damage indication, Figure 8 summarizes the complete results of an in situ tensile test in SEM by correlating observed microstructural changes to the applied tensile stress σ_T._ In Figure 8a, the σ–ε curve with marked points for SEM micrographs is displayed, whereas in Figure 8b, occurring side effects of SEM, like charging of the non-conductive material (I–III), as well as damage mechanisms (i–iii) are defined. The depicted SEM micrographs in Figure 8c correspond to the marked points on the σ–ε curve. From analysis of the surface in its initial condition (1), the impact of the focused electron beam on the cellulosic material in terms of charging effects is clearly visible. It is stated here that the presented results should give a first idea of the microstructural damage and failure behavior of Cottonid. How the results can be transferred into the macroscopic range and how the carbon coating on the surface for avoidance of charging effects of the non-conductive material influences the mechanical behavior has to be verified over ongoing studies. A first interpretation of the damage development is provided in the following.

With increasing tensile stress σ_T_, first cracks initiate at σ_T,2_ = 45.62 MPa and a nominal strain of ε_n,2_ = 0.52 × 10^−2^ at the end of the quasi elastic area (2, i) from imperfections, in this example, in the edge of the gauge length. Here, a significant influence of the specimen preparation prior to the experiment can be expected, but by passing the yield strength (3), cracks also initiate on the unprepared surface at σ_T,3_ = 61.85 and ε_n,3_ = 1.08 × 10^−2^. Further loading (4) leads to crack initiations at the upper rim at σ_T,4_ = 63.16 MPa and ε_n,4_ = 1.24 × 10^−2^. These cracks assemble within the plastic region (5–8), while another crack initiates from the opposite edge of the gauge length. After reaching the ultimate tensile strength (UTS) at σ_T,f_ = 70.84 MPa (ε_n,f_ = 2.31 × 10^−2^), these mechanisms lead to complete loss of structural integrity (9) and the assembly of all monitored cracks (10–12) to final failure of the specimen.

Damage development on Cottonid’s surface due to quasi-static tensile loading is therefore characterized by crack initiations on microstructural characteristics, like pores and microcracks, but also in amorphous areas. These findings correspond to studies by Liu et al. on fiber-reinforced polymer-derived ceramic composites [23], who also observed crack initiation at processing-induced voids and propagation after reaching the yield strength. A more precise surface preparation could improve the ultimate tensile strength, but it is challenging because of the natural roughness and hygroscopicity of Cottonid. Unlike the results of Arif [25] on damage development in PA66/GF30 composites, cracks propagate throughout the whole Cottonid structure, instead of growing along weaker areas, like fiber-matrix interfaces. Therefore, the prediction and modelling of the damage development on Cottonid’s surface is challenging [37,38].

### 3.2. In Situ Quasi-Static Tensile Tests in µCT

**Figure 9:**
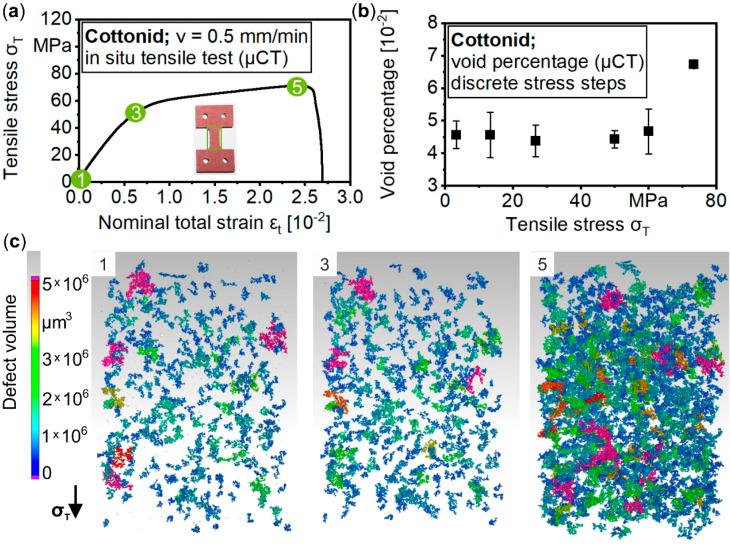
In situ tensile test in µCT: (**a**) significant points in stress–strain curve for performing µCT scans; (**b**) percentage development of void volume and (**c**) visualization of void volume in the specimen’s gauge length for discrete tensile load steps.

Figure 9 comprises the result of an in situ tensile test in µCT by correlating observed void volumes in the specimen’s gauge length to the applied tensile stress σ_T._ Because in situ µCT investigations are much more time-consuming compared to SEM, less tensile loads were investigated. Figure 9a displays the σ–ε curve with marked points for µCT scans, whereas in Figure 9b, the detected void volume percentage in relation to the initial state is plotted over discrete tensile stresses σ_T_. In its initial state (1), Cottonid already exhibits a great amount of voids, which could be explained by the manufacturing process, where several paper layers are layered over each other and bonded by a chemical process. Insufficiently bonded areas result in delaminations/voids within the bulk material. In the elastic region (2), the overall void volume first decreases until reaching the yield strength (3) because of deformation-induced closing of the voids. With further loading into the plastic region (4), the overall void volume increases until final failure of the specimen at σ_T,f_ = 70.84 MPa (ε_n,f_ = 2.31 × 10^−2^). These microstructural mechanisms are exemplarily visualized for three loading stages (Figure 9c). The different colours represent the maximum void volume, whereas the material itself is rendered invisible to focus on void development due to the mechanical loading.

A more detailed analysis of the percentage development of the single sections can be found in Table 2.

In correlation to the surface defects obtained in in situ SEM investigations, the void volume in the initial condition could be explained by insufficiencies in the material originating from the rough surface of the cellulose fibers as well as existing pores and microcracks (~10,000–1,000,000 µm^3^) and insufficiently bonded areas caused by the manufacturing process (>1,000,000 µm^3^). The propagation and assembly of these damage mechanisms have already been interpreted. A further delamination of the chemically bonded paper layers within the plastic region starting from insufficiently bonded areas in the initial condition is expected. This hypothesis correlates to µCT studies of Arif [25,39], also in which debonding of interfaces in a PA66/GF30 composite was identified as a damage mechanism due to a mechanical tensile load.

## 4. Conclusions and Outlook

Within this study, microstructural damage development on the surface and in the volume of the polymeric material Cottonid due to quasi-static tensile loading was assessed using advanced in situ scanning electron microscope and microfocus computer tomography techniques. Via optical monitoring of microstructural characteristics of the material in its initial condition, the progression of defects like pores, microcracks or delaminations could be correlated with applied tensile loads. Therefore, the deformation, damage and failure behavior of Cottonid could be interpreted for different regions of the stress–strain curve. In the elastic region, no visible damage occurs on the surface, whereas delaminations/voids present in the volume first decrease due to deformation-induced closing. When passing the yield strength, cracks initiate at critical areas on the surface (pores, microcracks) and in the volume (delaminations/voids) and propagate throughout the whole structure with increasing load. The assembly of these cracks leads to loss of structural integrity and final failure of the specimen. Corresponding to studies on fiber-reinforced composites [25,33], microcrack propagation starts after reaching the yield strength, but it is not characterized by known phenomena, like propagation along fiber–matrix interfaces. In fact, microcracks initiate variously on the surface and propagate by cutting fibers and amorphous areas equally. Furthermore, there is no dominant crack [21], which leads to failure, but all monitored cracks had an impact on the loss of structural integrity of the Cottonid specimen. Similar to laminated composites [31,32], present voids detected in the volume seem to increase within the interfaces between the paper layers, which macroscopically are not visible anymore after the chemical treatment during parchmentizing.

How the carbon coating on the surface for avoiding charging effects during the analytical investigations affects the mechanical behavior of Cottonid and how the results can be transferred into the macroscopic range has to be verified in ongoing studies. Furthermore, the impact of the direction-dependent deformation behavior, visible in the plastic region of the stress–strain curve, as well as temperature and humidity effects on the damage initiation and evolution during a mechanical loading will be investigated further.

## Figures and Tables

**Table 1:** Lead time before starting a µCT scan at different tensile loads to avoid formation of image artifacts.

Tensile Stress σ_T_ [MPa]	Lead Time t_s_ before µCT Scan [s]
13.3	57
26.6	60
50.0	1080
60.0	1580
73.3	2300

**Table 2:** Percentage development of void volume in the specimen’s gauge length at different tensile loads in an in situ tensile test.

Defect Volume [µm^3^]	Development of Void Volume in Relation to Initial Condition [%]				
>1,000,000	−7 ± 37	−16 ± 25	−9 ± 25	+2 ± 35	+418 ± 127
1,000,000–500,000	+3 ± 26	−5 ± 21	+2 ± 21	+10 ± 18	+169 ± 63
499,999–100,000	−1 ± 15	−5 ± 10	−2 ± 10	+4 ± 12	+56 ± 22
99,999–10,000	0 ± 8	−2 ± 7	−2 ± 7	−1 ± 5	+10 ± 8
<10,000	0 ± 11	−4 ± 9	−5 ± 10	−3 ± 7	+12 ± 5
Tensile stress σ_T_ [MPa]	13.3	26.7	50.0	60.0	73.3

## References

[1] Taylor T. (1871). Improvement in the Treatment of Paper and Paper-Pulp. U.S. Patent.

[2] Schoenen F., Vieweg R., Becker E. (1965). 3: Cellulose als Ausgangsstoff. Kunststoff Handbuch Band 3 Abgewandelte Naturstoffe Herstellung, Eigenschaften, Verarbeitung und Anwendungen.

[3] Ernst Krueger GmbH. & Co. KG. https://www.hornex.de/.

[4] Sachsenroeder GmbH. & Co. KG. https://sachsenroeder.com/.

[5] Scholz R., Mittendorf R.-M., Engels J.K., Hartmaier A., Kuenne B., Walther F. (2016). Direction-dependent mechanical characterization of cellulose-based composite vulcanized fiber. Mater. Test..

[6] Scholz R., Delp A., Kaplan A., Walther F., Christ H.-J. (2016). Vergleichende Bewertung der temperaturabhängigen mechanischen Eigenschaften von Vulkanfiber und technischen Kunststoffen. Werkstoffprüfung 2016-Fortschritte in der Werkstoffprüfung für Forschung und Praxis.

[7] Frey M., Widner D., Segmehl J.S., Casdorff K., Keplinger T., Burgert I. (2018). Delignified and densified cellulose bulk materials with excellent tensile properties for sustainable engineering. ACS AMI.

[8] Scholz R., Langhansl M., Zollfrank C., Walther F., Wiedemann M., Melz T. (2019). Cottonid-Ein effizienter Funktionswerkstoff für feuchtegetriebene Aktuatoren. Smarte Strukturen und Systeme-Tagungsband des 4 Smarts-Symposiums.

[9] Scholz R., Langhansl M., Zollfrank C., Walther F. (2019). Experimental study on the actuation and fatigue behavior of the biopolymeric material Cottonid. Mater. Today Proc..

[10] Poppinga S., Zollfrank C., Prucker O., Rühe J., Menges A., Cheng T., Speck T. (2018). Toward a new generation of smart biomimetic actuators for architecture. Adv. Mater..

[11] Awd M., Siddique S., Walther F. (2020). Microstructural damage and fracture mechanisms of selective laser melted Al-Si alloys under fatigue loading. Theor. App. Fract. Mec..

[12] Backe S., Balle F. (2018). A novel short-time concept for fatigue life estimation of carbon (CFRP) and metal/carbon fiber reinforced polymer (MCFRP). Int. J. Fatigue.

[13] Giovino M., Pribyl J., Benicewicz B., Bucinell R., Schadler L. (2019). Mechanical properties of polymer grafted nanoparticle composites. Nanocomposites.

[14] Mammadi Y., Joseph A., Joulain A., Boneeville J., Tromas C., Hedan S., Valle V. (2020). Nanometric metrology by FIB-SEM-DIC measuremtns of strain field and fracture separation on composite metallic material. Mater. Des..

[15] Hülsbusch D., Kohl A., Striemann P., Niedermeier M., Strauch J., Walther F. (2020). Development of an energy-based approach for optimized frequency selection for fatigue testing on polymers—Exemplified on polyamide 6. Polym. Test..

[16] Mao H., Xiao X.Y., Mao H., Tang W., Huang Z., Li X., Sun L. (2019). Fatigue damage detection and location of metal materials by electrical impedance tomography. Results Phys..

[17] Tillmann W., Walther F., Luo W., Haack M., Nellesen J., Knyazeva M. (2018). In Situ Acoustic Monitoring of Thermal Spray Process Using High-Frequency Impulse Measurements. J. Therm. Spray Technol..

[18] Ivanov S.G., Gorbatikh L., Lomov S.V., Verpoest I. Textile composites in tension: In situ observation of damage development. Proceedings of the Composites Week @ Leuven and TexComp-11 Conference.

[19] Myslicki S., Ortlieb M., Frieling G., Walther F. (2015). High-precision deformation and damage development assessment of composite materials by high-speed camera, high-frequency impulse and digital image correlation techniques. Mater. Test..

[20] Gerbe S., Tenkamp J., Scherbring S., Bleicher K., Krupp U., Michels W., Walther F. (2019). Microstructural influences on the fatigue crack initiation and propagation mechanisms in hypo-eutectic Al-Si cast alloys. Proc. Struct. Integ..

[21] Kucharczyk P., Sharaf M., Münstermann S. (2012). On the influence of steel microstructure on short crack growth under cyclic loading. Int. J. Fatigue.

[22] Niederberger C., Mook W.M., Maeder X., Michler J. (2010). In situ electron backscatter diffraction (EBSD) during the compression of micropillars. Mater. Sci. Eng. A.

[23] Liu Y., Tanaka Y. (2003). In situ characterization of tensile damage behavior of a plain-woven fiber-reinforced polymer-derived ceramic composite. Mater. Lett..

[24] Scott A.E., Mavrogordato M., Wright P., Sinclair I., Spearing S.M. (2011). In situ fibre fracture measurement in carbon-epoxy laminates using highresolution computed tomography. Compos. Sci. Technol..

[25] Arif M.F., Meraghni F., Chemisky Y., Despringre N., Robert G. (2014). In situ damage mechanisms investigation of PA66/GF30 composite: Effect of relative humidity. Compos. Part B Eng..

[26] Hülsbusch D., Mrzljak S., Walther F. (2019). In situ computed tomography for the characterization of the fatigue damage development in glass fiber-reinforced polyurethane. Mater. Test..

[27] Schilling P.J., Karedla B.P.R., Tatiparthi A.K., Verges M.A., Herrington P.D. (2005). X-ray computed microtomography of internal damage in fiber reinforced polymer matrix composites. Compos. Sci. Technol..

[28] Little J.E., Yuan X., Jones M.I. (2012). Characterisation of voids in fibre reinforced composite materials. NDT&E Int..

[29] Yu B., Bradley R., Soutis C., Hogg P., Withers P. (2015). 2D and 3D imaging of fatigue failure mechanisms of 3D woven composites. Compos. Part A.

[30] Patera A., Derome D., Griffa M., Carmeliet J. (2013). Hysteresis in swelling and in sorption of wood tissue. J. Struct. Biol..

[31] Hamad W.H., Provan J.W. (1995). Microstructural cumulative material degradation and fatigue-failure micromechanisms in wodd-pulp fibres. Cellulose.

[32] Adey-Johnson R., Mclean J.P., Van den Bulcke J., Van Acker J., McDonald P.J. (2020). Micro-CT measurements of within-ring variability in longitudinal hydraulic pathways in Norway spruce. IAWA J..

[33] Barbero E.J., Cosso F.A., Campo F.A. (2013). Benchmark solution for degradation of elastic properties due to transverse matrix cracking in laminated composites. Comp. Struct..

[34] Carraro P.A., Quaresimin M. (2015). A stiffness degradation model for cracked multidirectional laminates with cracks in multiple layers. Int. J. Solids Struct..

[35] Reifsnider K., Raihan M.R., Vadlamudi V. (2016). Heterogeneous fracture mechanics for multi defect analysis. Compos. Struct..

[36] G’sell C., Dahoun A. (1994). Evolution of microstructure in semi-crystalline polymers under large plastic deformation. Mater. Sci. Eng. A.

[37] Landis E.N., Vasic S., Davids W.G., Parrod P. (2002). Coupled experiments and simulations of microstructural damage in wood. Exp. Mech..

[38] Qing H., Mishnaevsky L.J. (2011). A 3D multilevel model of damage and strength of wood: Analysis of microstructural effects. Mech. Mater..

[39] Chaboche J.L., Girard R., Levasseur P. (1997). On the interface deponding models. Int. J. Damage Mech..

